# Improving the evaluation of COPD exacerbation treatment effects by accounting for early treatment discontinuations: a post-hoc analysis of randomized clinical trials

**DOI:** 10.1186/s12931-020-01419-8

**Published:** 2020-06-22

**Authors:** Agnieszka Król, Robert Palmér, Virginie Rondeau, Stephen Rennard, Ulf G. Eriksson, Alexandra Jauhiainen

**Affiliations:** 1 Clinical Pharmacology and Safety Sciences, BioPharmaceuticals R&D, AstraZeneca, Gothenburg, Sweden; 2 Biostatistics Team, INSERM CR1219, University of Bordeaux, Bordeaux, France; 3 BioPharmaceuticals R&D, AstraZeneca, Cambridge, UK; 4 University of Nebraska Medical Center, Omaha, NE USA; 5 BioPharma Early Biometrics and Statistical Innovation, Data Science & AI, BioPharmaceuticals R&D, AstraZeneca, Pepparedsleden 1, SE-431 83 Mölndal, Sweden

**Keywords:** COPD, Early treatment discontinuations, Dropouts, Exacerbations, Joint frailty model, Recurrent events, Survival analysis

## Abstract

**Background:**

Chronic obstructive pulmonary disease (COPD) clinical trials aimed at evaluating treatment effects on exacerbations often suffer from early discontinuations of randomized treatment. Treatment discontinuations imply a loss of information and should ideally be considered in the statistical analysis of trial results, particularly if the discontinuations are related to the disease or treatment itself. Here, we explore this issue by investigating (1) whether there exists an association between the risks of exacerbation and treatment discontinuation in COPD clinical trials and (2) whether disregarding this association can cause bias in exacerbation treatment effect estimates. We focus on the hypothetical estimand, i.e. the treatment effect that would have been observed had all subjects completed the trial as planned.

**Methods:**

The association between exacerbation and discontinuation risks was analysed by applying a joint frailty (random effect) model – allowing for the simultaneous analysis of multiple types of correlated events – to data from five Phase III-IV COPD clinical trials. Specifically, the impact of the association on exacerbation treatment effect estimates was assessed by comparing the treatment hazard ratios of the joint frailty model to the rate/hazard ratios of two related statistical models (the negative binomial and shared frailty models), which both assume discontinuations to be unrelated to the trial outcome. The models were also compared using simulated data.

**Results:**

A statistically significant (*p* < 0.0001), positive association between exacerbation and discontinuation risks was found in all trials. Importantly, simulations confirmed that – with such an association – models disregarding the association risk producing biased results (> 5 percentage point difference in hazard/rate ratio). For some treatment comparisons in the clinical trials, the difference in treatment effect estimates between the joint frailty and the other models was as high as 10–15 percentage points. The difference was affected by the strength of the exacerbation-discontinuation association, the population heterogeneity in exacerbation risk, and the difference in discontinuation rates between treatment arms.

**Conclusions:**

We have identified an association between the risks of exacerbation and treatment discontinuation in five COPD clinical trials. We recommend using the joint frailty model to account for this association when estimating exacerbation treatment effects, particularly when targeting the hypothetical estimand.

## Background

In late-phase, randomized chronic obstructive pulmonary disease (COPD) clinical trials, a significant number of patients often discontinue their randomized treatment before the planned end of the trial [1, 2]. Many of these early treatment discontinuations can be associated to e.g. disease worsening or adverse events and thus be considered as informative censoring events. As such, they may influence the estimation of treatment effects and lead to biased trial outcomes if not handled properly [3–6].

Herein, we investigate the impact of early treatment discontinuations on COPD exacerbation treatment effect estimates. COPD exacerbations are often used as a key endpoint in late-phase COPD clinical trials. However, the appropriate statistical approach used to analyse them is a topic of scientific debate [7–11]. Typically, exacerbations are analysed either as a rate endpoint using negative binomial regression or as a time-to-first event endpoint using the Cox proportional hazards model [12–14], but both these approaches have limitations.

Particularly in longer clinical trials (≥6 months), where patients may experience multiple exacerbations, information may be lost if only the time-to-first exacerbation is used as in a standard Cox analysis. Moreover, the Cox analysis typically neglects patient heterogeneity in exacerbation risk and assumes that, given the same set of covariates (e.g. treatment and potential prognostic factors), the risk for each patient is the same. The negative binomial model, on the other hand, can handle both multiple events per subject and unobserved risk heterogeneity but instead assumes constant risk over time and ignores the timing and ordering of events.

One way to alleviate some of the assumptions of standard methods would be to use the shared frailty model [15]. The shared frailty model is based on the Andersen-Gill (AG) counting process model, a generalization of the Cox model for the analysis of recurrent events [16]. Extending the AG model to the shared frailty model is done by introducing a random effect called *frailty*. Not unlike its medical interpretation, the term *frailty* is used to indicate that some individuals may have a higher risk of an event (in our case exacerbations) than others. That is, like the random effect in the negative binomial model, the *frailty* describes risk heterogeneity and allow for the analysis of correlated recurrent events within individuals in a flexible way. Importantly, though, neither the shared frailty nor standard models address the issue of informative censoring. Instead, the assumption that discontinuations are unrelated to the trial outcome, i.e. that exacerbation data are missing at random (MAR), is often used with these models when the aim is to estimate the treatment effect as if all subjects would have completed the trial as planned, i.e. when targeting the de jure or hypothetical estimand [10, 17, 18].

Keene et al. [10, 19] have previously suggested using various imputation methods to study the sensitivity of exacerbation treatment effect estimates to the MAR assumption, focusing on the negative binomial model and treatment policy estimand [17]. Here, however, we consider a novel approach to particularly address the hypothetical estimand. This approach involves the use of a joint frailty model [20, 21] for the simultaneous analysis of recurrent episodes of moderate/severe exacerbations and early treatment discontinuations as two semi-competing, possibly correlated, types of events. Specifically, we hypothesize that there exists an association between a patient’s risk of exacerbation and risk early treatment discontinuation and that disregarding this association in the statistical analysis may lead to biased treatment effect estimates. To investigate this hypothesis, we perform a post-hoc analysis and compare the treatment hazard ratios of the joint frailty model to the hazard/rate ratios of the shared frailty and negative binomial models using both simulated data and patient-level data from five Phase III-IV clinical trials in moderate-to-severe COPD patients. We also use the clinical trial data to identify and adjust the joint frailty model with key prognostic factors of exacerbation and discontinuation risks.

## Methods

### Clinical trial datasets

Patient-level data from five Phase III-IV, randomized, double-blind, parallel-group, multicentre trials including a total of 7698 patients with moderate-to-severe COPD served as the basis for this post-hoc analysis (Table 1). The trials were selected to be long and large enough to provide reasonable numbers of early treatment discontinuations and exacerbations to enable appropriate analysis of the association between them (moderate/severe exacerbations were included as either a primary or secondary endpoint in all trials). From the perspective of this analysis, there were no important differences in the design of the Phase III and Phase IV trials.

**Table 1:** Summary of clinical trial datasets

Dataset	N (original)	N (analysis)	Duration (months)	Treatment groups*	Endpoints
A	1964	1746	12	1) BUD/FM 320 2) BUD/FM 160 3) FM 4) **PBO**	Primary: pre- and post-dose FEV_1_ Secondary: number of exacerbations
B	1219	1072	12	1) BUD/FM 320 2) BUD/FM 160 3) **FM**	Primary: number of exacerbations
C	1704	1613	6	1) BUD/FM 320 2) BUD/FM 160 3) BUD 320 + FM 4) BUD 320 5) FM 6) **PBO**	Primary: pre- and post-dose FEV_1_ Secondary: number of exacerbations
D	1945	1218	12	1) SOC + RFL 2) **SOC**	Primary: rate of exacerbations
E	2354	2049	12	1) SOC + RFL 2) **SOC**	Primary: rate of exacerbations

*BUD* budesonide, *FEV*_*1*_ forced expiratory volume in 1 s, *FM* formoterol, *PBO* placebo, *RFL* roflumilast, *SOC* standard of care, * - Treatment groups: BUD/FM 320 - BUD/FM pMDI (pressurized metered-dose inhaler) 160/4.5 μg × 2 inhalations bid (twice daily) (320/9 μg), BUD/FM 160 - BUD/FM pMDI 80/4.5 μg × 2 inhalations bid (160/9 μg), FM - FM DPI (dry powder inhaler) 4.5 mcg × 2 inhalations bid (9 μg), BUD 320 - BUD pMDI 160 μg × 2 inhalations bid (320 μg), BUD 320 + FM - BUD pMDI 160 μg × 2 inhalations bid (320 μg) and FM DPI 4.5 μg × 2 inhalations bid (9 μg), PBO - use of SABA (short-acting beta agonists) only, SOC - standard of care with inhaled corticosteroid and long-acting beta agonist +/− long-acting muscarinic receptor agonist, SOC + RFL - standard of care and RFL 500 μg once daily. The reference treatment in each dataset is marked in bold

Trials A [22], B [23], and C [24] were of 6–12 months duration and evaluated the efficacy of combinations of budesonide (inhaled corticosteroid, ICS) and formoterol (inhaled long-acting beta agonist, LABA) in moderate-to-severe COPD patients. Trials D [13] and E [14] were both of 12 months duration and evaluated the efficacy of roflumilast (oral phosphodiesterase 4 inhibitor) on top of standard of care (SOC) treatment with ICS and LABA, plus/minus a long-acting muscarinic receptor antagonist (LAMA), in patients with severe COPD. All trials were conducted in accordance with the Declaration of Helsinki, the International Conference on Harmonisation Guidelines for Good Clinical Practice, and applicable regulatory requirements. For further details on the clinical trials, see Additional file 1 and the original trial publications.

Before the analysis, all informed consent forms were reviewed for data re-use in accordance with AstraZeneca data sharing rules. Patients from countries where ethics committees did not approve data re-use, as well as patients who had withdrawn consent, were excluded. Consequently, only a subset of the original trial data were part of this analysis and any direct comparison with the original trial results should be made with care. We refer to the subsets of data as Datasets A-E (see Table 1 for a comparison of the number of patients included in the original trials and in this analysis).

### Definition of exacerbations and early discontinuations

A COPD exacerbation was defined as an episode of worsening of respiratory symptoms requiring additional treatment with oral corticosteroids, antibiotics, and/or hospitalization (i.e. moderate/severe exacerbations). The start and end time of an exacerbation was defined as the number of days from randomization to first initiation and last cessation of additional treatments, respectively. Patients were considered not at risk during an exacerbation episode.

Early treatment discontinuations were defined as any discontinuation of randomized treatment before the planned end of treatment day. The time of discontinuation was defined as the day of the last dose of the randomized treatment. End-of-trial completion was treated as a non-informative right-censoring event. Reasons for early treatment discontinuations (to the extent known) in the different studies are summarised in Additional file 1**: Table S1**.

### Statistical analysis

Statistical analyses were carried out using R version 3.4.3 [25] (packages listed in Additional file 1).

**Fig. 1:**
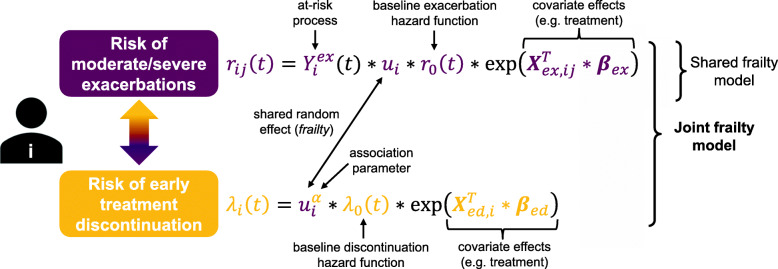
Outline of the joint frailty model for recurrent exacerbations and early treatment discontinuation risks. Baseline hazard functions are denoted by *r*_0_(*t*) and *λ*_0_(*t*) for exacerbations and early treatment discontinuations, respectively. The frailty *u*_*i*_ is a random effect from a gamma distribution and *α* represents the strength of the association between exacerbations and discontinuations. Vectors ***X***_*ex*, *ij*_ and ***X***_*ed*, *i*_ denote covariates related to exacerbations and early discontinuations, respectively, ***β***_*ex*_ and ***β***_*ed*_ are the corresponding regression coefficients. The at-risk process for exacerbations (subjects not at risk during an ongoing exacerbation episode) is denoted ${Y}_i^{ex}(t)$

Recurrent episodes of moderate/severe exacerbations and early treatment discontinuations were simultaneously analysed using a joint frailty model [26], at first with treatment as the only covariate (for both exacerbations and discontinuations). The model (Fig. 1) consists of two sub-models – an Andersen-Gill model for recurrent episodes of exacerbations and a time-to-first event proportional hazards model for early discontinuations – linked using a gamma-distributed random effect (*frailty*). The *frailty* describes the between-patient variability in exacerbation (and discontinuation) risk and acts proportionally on the baseline hazards, which are approximated with splines allowing for time-varying risk. A *frailty* variance of zero would indicate that all subjects (with the same set of covariates) have the same risk of experiencing an event, while an increasing *frailty* variance implies an increasing between-patient difference in risk, as well as an increasing difference in the number of events per subject. In the discontinuation hazard function, the *frailty* is scaled by an exponent (α) describing the strength of the association between discontinuation and exacerbation risks. The value of α is valid if the estimated variance of the *frailty* is significantly greater than zero, with the interpretation that for α > 0 and α < 0 the risks are positively and negatively associated, respectively. If α = 0, the risks are considered unrelated, and the joint frailty model reduces to the shared frailty model (for the exacerbation part, Fig. 1). Wald-tests were used to test if α ≠ 0 and *frailty* variance > 0. For more details, see Additional file 1**.**

Firstly, the impact of disregarding an association between exacerbation and treatment discontinuation risks was investigated using simulated data from the joint frailty model. Simulating data allowed us to investigate – in a controlled way – what properties of exacerbation and discontinuation data may cause biased treatment effect estimates. The simulated data included two treatment groups (control and active) with overall exacerbation and discontinuation rates, as well as number of patients, chosen to be similar to what we observed in Datasets A-E. Several scenarios were evaluated: 1) different strengths of the risk association, 2) different levels of *frailty* variance, and 3) different discontinuation rates in the two treatment groups.

After simulating data (1000 datasets per scenario), the effect of disregarding the association in the statistical analysis was evaluated by fitting a shared frailty model to the data and comparing the estimated exacerbation hazard ratios to the “true” hazard ratio used in the simulations. The joint frailty model itself was also fitted to the data for comparison, to make sure it produced unbiased estimates of the hazard ratio. See Additional file 1 for further information on how the simulation study was done.

Secondly, the joint frailty model was applied to the clinical trial data (Datasets A-E) and exacerbation hazard ratio estimates of the model were compared to the hazard/rate ratios of both the shared frailty and negative binomial models. Hazard/rate ratios were calculated using the original comparator arm in each trial as reference, i.e. placebo in Datasets A and C, formoterol in Dataset B, and SOC in Datasets D and E (Table 1). We decided not to include a comparison with a standard time-to-first-event Cox analysis, since the focus of this work is on the impact of treatment discontinuations and not on the comparison of time-to-first-event and recurrent-event analyses.

Finally, the joint frailty model was further evaluated by adjusting for prognostic factors of exacerbation and discontinuation risks and evaluating their impact on the *frailty* variance and risk association. Prognostic factors were selected based on an extensive covariate search using pooled data from Datasets A-C and D-E (see Additional file 1 for details). First, LASSO Cox regression [27] was performed separately on exacerbation and discontinuation data. The most frequently selected covariates were then complemented with other common prognostic factors, and stepwise backwards selection using the joint frailty model was performed. The tested covariates included patient demographic and disease history data, as well as baseline measures of clinical lab variables, spirometry variables, and patient reported outcomes (PROs), such as diary recordings, St. George’s Questionnaire (SGRQ), and the COPD Assessment Test™ (CAT). Seasonality – a known predictor of exacerbation risk [28] – was also added to the model as a time-varying covariate.

## Results

### Clinical data characteristics

Exacerbation and discontinuation data for Datasets A-E are summarised in Table 2. The percentage of patients with at least one moderate/severe exacerbation and the overall number of moderate/severe exacerbations per patient time on treatment varied between 28 and 55% and 0.816–1.293 events/year, respectively. Early treatment discontinuation frequencies were between 15 and 23%.

**Table 2:** Summary data of analysis datasets with respect to recurrent exacerbations and early discontinuations

	Dataset A	Dataset B	Dataset C	Dataset D	Dataset E
Number of patients available for analysis	1746	1072	1613	1218	2049
Number of mod/sev exacerbations	1186	949	655	1350	2179
Number of patients with R number of mod/sev exacerbations					
R = 0	1086	537	1170	604	920
R = 1	376	286	301	265	554
R ≥ 2	284	249	142	349	575
Percentage of patients with ≥1 mod/sev exacerbation	37.8%	49.9%	27.5%	50.4%	55.1%
Max. number of mod/sev exacerbations	12	10	6	11	11
Mean number of mod/sev exacerbations per patient years at risk	0.816	1.051	0.917	1.284	1.293
Number of early treatment discontinuations	392	221	235	180	421
Percentage of early treatment discontinuations	22.5%	20.6%	14.6%	14.8%	20.5%
Percentage of early treatment discontinuations in original data	31%	30%	19%	24%	25%

*mod* moderate, *sev* severe

Table 2 also shows the discontinuation frequencies in the original trials. As can be seen, original frequencies are higher than in our datasets, indicating that many of the patients who had to be excluded in this analysis, due to data re-use rules, were patients who also had discontinued treatment.

**Fig. 2:**
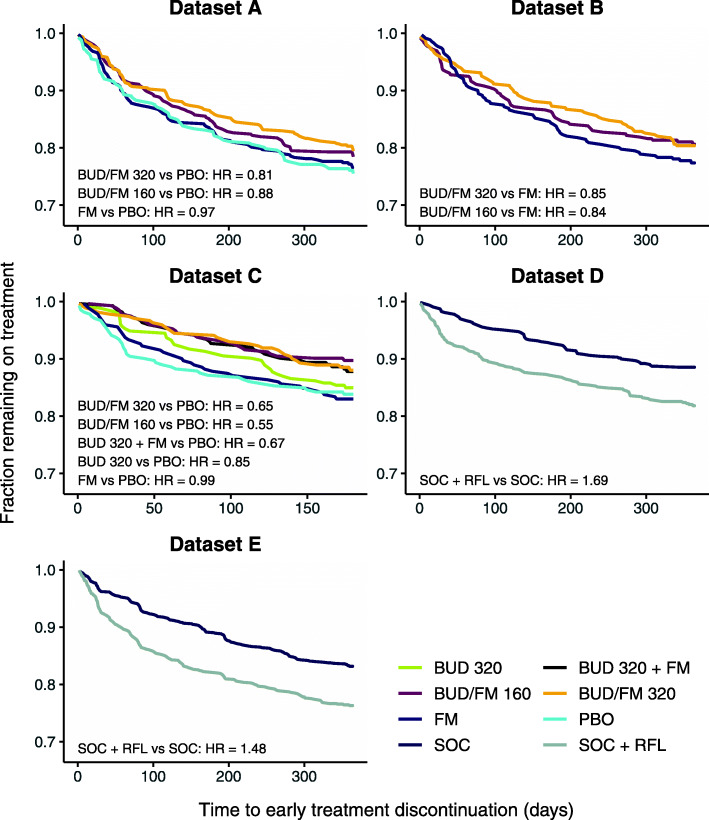
Kaplan-Meier curves for time to early treatment discontinuation for each treatment arm in datasets A-E. The hazard ratio (HR, from a proportional hazards model) is given for the relevant comparisons in each dataset. BUD - budesonide, FM - formoterol, PBO - placebo, RFL - roflumilast, SOC - standard of care

Kaplan-Meier curves of early treatment discontinuations for each dataset and treatment arm are summarised in Fig. 2 together with corresponding hazard ratios. In Datasets A-C, treatments considered more effective in terms of disease control (combinations of budesonide and formoterol) were associated with a reduced risk of early treatment discontinuation compared to less effective treatments arms (formoterol or placebo), with discontinuation hazard ratios between 0.55–0.99. In Datasets D-E, on the other hand, higher rates of early treatment discontinuations were seen in the roflumilast arms compared to SOC treatment alone (discontinuation hazard ratios 1.48 and 1.69).

### Analysis of simulated data

**Fig. 3:**
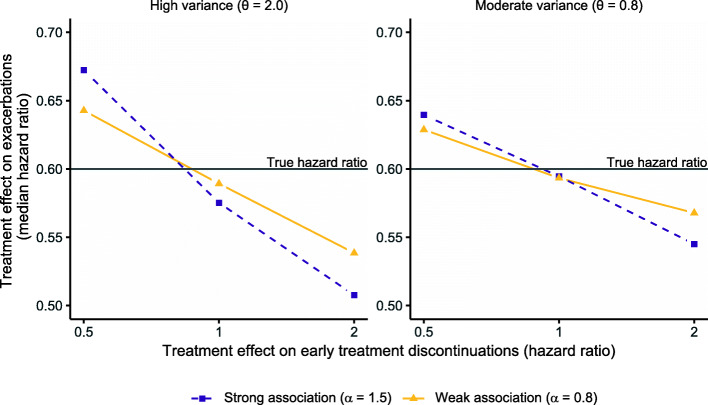
Hazard ratios for exacerbations estimated using a shared frailty model fitted to simulated data. Data were generated using a joint model with a gamma-distributed frailty with different levels of frailty variance, different association between exacerbation and discontinuation risks, and different treatment discontinuation rates between the two treatment arms. The mean (overall) rate of exacerbations was set to 0.9 per year, the overall percentage of discontinuations to 25%, and the true hazard ratio for the risk of exacerbations to 0.6 in all scenarios. The y-axis shows the estimated median hazard ratio across 1000 simulations

The parameters used for simulating exacerbation and discontinuation data are described in Fig. 3. The figure also illustrates the difference (bias) between the “true” and estimated exacerbation treatment effect of a shared frailty model when applied to the simulated data. The shared frailty model was found to underestimate the treatment effect with a difference in hazard ratio of ~ 7 percentage points (0.67 compared to 0.6) in cases of high *frailty* variance, a strong risk association, and a protective treatment effect on treatment discontinuations (discontinuation hazard ratio = 0.5). Conversely, the treatment effect was overestimated if active treatment caused more discontinuations than the control arm (discontinuation hazard ratio = 2). A tendency of overestimation was also seen when there was no difference in discontinuation rate between the two groups. Overall, the bias got smaller with smaller *frailty* variance and weaker risk association.

Results using other exacerbation and treatment discontinuations rates are found in Additional file 1: **Figure S1**, highlighting that the overall treatment discontinuation rate in a trial is also an important factor affecting the bias (increased rate leads to increased bias). Using the joint frailty model to estimate the hazard ratio resulted in no bias (Additional file 1: **Figure S2**).

### Analysis of clinical trial data

Statistically significant *frailty* variances and exacerbation-discontinuation associations was found in all clinical trial datasets (*p* < 0.0001 for both parameters in all datasets, Table 3). The parameter estimates for the *frailty* variance and association varied between 0.74–2.17 and 0.83–1.84, respectively. The lowest association was seen in the two roflumilast datasets (Datasets D-E).

**Table 3:** Estimates of the association parameters for recurrent exacerbations and discontinuations using the joint frailty model

	Frailty variance	Association (*α*)
	Estimate (SE)*	
Dataset A	1.84 (0.13)	0.99 (0.11)
Dataset B	0.87 (0.10)	1.84 (0.24)
Dataset C	2.17 (0.20)	1.28 (0.23)
Dataset D	1.34 (0.11)	0.93 (0.18)
Dataset E	0.74 (0.06)	0.83 (0.16)

*SE* standard error, * - all parameters significantly different from zero with *p*-value < 0.0001

A comparison of the estimated exacerbation hazard ratios of the joint frailty model with the hazard/rate ratios of the shared frailty and negative binomial models is shown in Table 4. In summary, the joint frailty model consistently estimated larger treatment effects than the other models for treatments with an early protective effect on treatment discontinuations, and smaller treatment effects in the reverse situation. The shared frailty and negative binomial models produced similar estimates across all datasets and treatment arms.

**Table 4:** Estimated exacerbation treatment effects and 95% confidence intervals for the different models in Datasets A-E

	Joint frailty model	Shared frailty model	Negative binomial model
	Exacerbation treatment ratio (95% confidence interval)*		
Dataset A			
BUD/FM 320 vs PBO	0.59 (0.45–0.77)	0.63 (0.49–0.81)	0.63 (0.49–0.80)
BUD/FM 160 vs PBO	0.58 (0.44–0.75)	0.60 (0.46–0.77)	0.60 (0.47–0.77)
FM vs PBO	0.90 (0.69–1.17)	0.90 (0.70–1.16)	0.90 (0.71–1.14)
Dataset B			
BUD/FM 320 vs FM	0.67 (0.54–0.82)	0.68 (0.55–0.83)	0.68 (0.55–0.83)
BUD/FM 160 vs FM	0.74 (0.60–0.92)	0.74 (0.60–0.90)	0.73 (0.59–0.89)
Dataset C			
BUD/FM 320 vs PBO	0.73 (0.50–1.08)	0.87 (0.60–1.25)	0.88 (0.62–1.24)
BUD/FM 160 vs PBO	0.70 (0.47–1.04)	0.83 (0.58–1.20)	0.84 (0.59–1.20)
BUD 320 + FM vs PBO	0.57 (0.38–0.85)	0.67 (0.46–0.98)	0.68 (0.47–0.98)
BUD 320 vs PBO	0.72 (0.48–1.07)	0.81 (0.55–1.18)	0.82 (0.57–1.17)
FM vs PBO	1.09 (0.74–1.61)	1.15 (0.80–1.66)	1.16 (0.82–1.63)
Dataset D			
SOC + RFL vs SOC	0.82 (0.68–0.98)	0.76 (0.64–0.90)	0.76 (0.64–0.90)
Dataset E			
SOC + RFL vs SOC	0.95 (0.85–1.07)	0.91 (0.81–1.02)	0.91 (0.81–1.02)

*BUD* budesonide, *FM* formoterol, *PBO* placebo, *RFL* roflumilast, *SOC* standard of care * - Hazard ratios are estimated for the joint and shared frailty models. A rate ratio is estimated for the negative binomial model. Only treatment is included as a covariate (for the joint frailty model, a treatment effect is included both in the exacerbation and discontinuation hazard)

For Datasets A-C, the difference in hazard/rate ratios between the joint frailty and the other models ranged between 0 and 15 percentage points, with the most pronounced difference seen in Dataset C. Of note is that, in the one case where the joint frailty model did not estimate a larger treatment effect (formoterol/budesonide 160 μg, Dataset B), there is a higher number of very early treatment discontinuations in the treatment arm compared to the reference (formoterol only) arm (Fig. 2).

For Dataset D-E, the joint model estimated smaller treatment effects than the shared frailty and negative binomial models, with a difference in hazard/rate ratio of 4–6 percentage points. This is in line with the discontinuation patterns seen in these datasets (Fig. 2), with roflumilast-treated patient having an increased treatment discontinuation rate.

Additional parameter estimates, such as the *frailty* variance and dispersion in the shared and negative binomial models, and the discontinuation hazard ratios in joint models, can be found in Additional file 1: **Tables S2-S3**.

### Covariate search

Three disease factors common to pooled Datasets A-C and D-E were found to be significantly associated with exacerbation risk in the covariate search: number of exacerbations in previous year, baseline FEV_1_, and baseline reliever medication use (Table 5). In addition, previous ICS and/or LABA use, smoking history, baseline breathlessness score, and baseline SGRQ score were identified as statistically significant covariates in pooled Dataset A-C, while baseline CAT score was identified as an influential covariate in pooled Dataset D-E. Geographic region was also found to be associated with exacerbation risk in both pooled datasets, with a higher risk of exacerbations in the US and Western Europe compared to other parts of the world. Adding seasonality as a time-varying covariate indicated that exacerbation risk varies significantly with season, with a ~ 30% lower risk during spring and summer compared to autumn and winter.

**Table 5:** Results from the joint frailty model, including selected covariates, when applied to the pooled datasets

	Exacerbations (moderate/severe)		Early treatment discontinuations	
	Pooled A-C HR (95% CI)	Pooled D-E HR (95% CI)	Pooled A-C HR (95% CI)	Pooled D-E HR (95% CI)
**Demographics**				
Age (+ 1 year)	1.00 (0.99–1.00)	1.00 (0.99–1.00)	1.01 (1.00–1.02)	1.01 (1.00–1.03)
Sex (male vs female)			1.29 (1.06–1.56)	1.24 (1.00–1.55)
**Region**				
Western Europe vs US	1.10 (0.91–1.34)	1.56 (1.28–1.90)	0.59 (0.43–0.82)	1.27 (0.86–1.87)
Eastern Europe vs US	0.49 (0.42–0.57)	0.61 (0.52–0.71)	0.21 (0.16–0.28)	0.45 (0.34–0.61)
Rest of the World vs US	0.83 (0.70–0.99)	0.83 (0.73–0.95)	0.30 (0.22–0.41)	0.50 (0.39–0.64)
**Treatment**				
BUD/FM 320 vs FM	0.71 (0.61–0.82)		0.73 (0.56–0.94)	
BUD/FM 160 vs FM	0.71 (0.61–0.83)		0.75 (0.59–0.97)	
BUD 320 + FM vs FM	0.57 (0.42–0.77)		0.58 (0.36–0.94)	
BUD 320 vs FM	0.77 (0.58–1.04)		1.03 (0.65–1.64)	
PBO vs FM	1.20 (1.00–1.44)		1.12 (0.84–1.50)	
SOC + RFL vs SOC		0.88 (0.80–0.97)		1.61 (1.34–1.94)
**Disease related baseline factors**				
Exacerbation history (+ 1 previous year)	1.16 (1.12–1.21)	1.21 (1.14–1.28)		
Smoking history (+ 10 pack-years)	1.02 (1.01–1.04)			
ICS history (yes vs no)	1.19 (1.06–1.34)			
Bronchodilator history (yes vs no)	1.30 (1.16–1.46)			
FEV_1_ (+ 100 mL)	0.92 (0.90–0.93)	0.52 (0.45–0.61)	0.7 (0.53–0.93)	0.551 (0.40–0.76)
SGRQ total score (+ 10 points)	1.04 (1.00–1.09)		1.01 (1.00–1.02)	
Breathlessness (+ 1 point)	1.11 (1.02–1.21)			
CAT total score (+ 5 points)		1.09 (1.05–1.13)		1.02 (1.00–1.03)
Use of rescue medication (+ 1 puff)	1.04 (1.03–1.06)	1.03 (1.02–1.04)	1.04 (1.01–1.06)	
**Seasonality**				
Spring vs Autumn	0.76 (0.68–0.85)	1.00 (0.90–1.11)		
Summer vs Autumn	0.69 (0.61–0.77)	0.78 (0.70–0.87)		
Winter vs Autumn	1.07 (0.97–1.19)	1.17 (1.06–1.29)		
**Trial effect**				
A vs C	0.76 (0.65–0.89)		0.86 (0.67–1.10)	
B vs C	0.85 (0.70–1.03)		0.78 (0.57–1.07)	
E vs D		1.08 (0.94–1.25)		1.64 (1.20–2.24)
	Association parameters*			
	**Pooled A-C**		**Pooled D-E**	
Frailty variance	1.14 (SE = 0.07)		0.67 (SE = 0.05)	
Association (α)	1.54 (SE = 0.15)		0.95 (SE = 0.17)	

*BUD* budesonide, *FM* formoterol, *RFL* roflumilast, *ICS* inhaled corticosteroids, *HR* hazard ratio, *CI* confidence interval, *SE* standard error, *US* United States, * - all parameters significantly different from zero with *p*-value < 0.0001

For early treatment discontinuations, age, sex, geographic region, baseline FEV_1_, baseline SGRQ score, baseline CAT score, and baseline reliever medication use were – in addition to treatment – identified as statistically significant covariates in one or both pooled datasets (Table 5). Noteworthy is that, even after including the covariates for both exacerbations and discontinuations, the remaining unexplained risk heterogeneity (*frailty* variance) was still statistically significant and only about 30% lower than in the corresponding models without covariates (Table 5**vs.** Additional file 1: **Table S4**). All tested and included covariates are summarised in Additional file 1: **Tables S5-S6** and **Figure S3**. Exacerbation and early treatment discontinuation baseline hazard functions are illustrated in Additional file 1: **Figure S4.**

## Discussion

Using a joint frailty model, we have identified a significant association between exacerbation and early treatment discontinuation risks in data from five Phase III-IV COPD clinical trials. Importantly, we show that ignoring this association in the statistical analysis of exacerbation treatment effects risks leading to substantially biased results, particularly when targeting the hypothetical estimand. Since biased statistical results may lead to incorrect conclusions regarding the effectiveness of treatments, we consider it important to thoroughly investigate this issue in future analyses of COPD clinical trials (e.g. by using the joint frailty model).

It is known that early treatment discontinuations can constitute an important source of bias in the estimates of treatment effects in clinical trials [4]. Patients with more severe disease, or patients experiencing lack of treatment effect or adverse events, may have an increased tendency to discontinue treatment [29, 30]. Interestingly, we found that regardless of the actual treatment effect on discontinuations, there is an underlying positive association between discontinuation and exacerbation risks in COPD trials. Specifically, we see that irrespectively of analysing combinations of budesonide and formoterol (shown to reduce treatment discontinuation rates compared to single treatment or placebo), or roflumilast (shown to increase treatment discontinuation rates, primarily due to gastrointestinal (GI) adverse events) [30], the association between risks – after adjusting for treatment effects – is statistically significant.

As shown in our simulation analysis, the size of the bias in exacerbation treatment effect estimates – caused by disregarding the association with treatment discontinuations – can be affected by multiple factors: the strength of the association, the size of the *frailty* variance (risk heterogeneity), and the treatment discontinuation rates in the different treatment arms. Moreover, the direction of the bias is determined by whether there is a higher (treatment effect is underestimated) or lower (treatment effect is overestimated) rate of discontinuations in the control/reference arm compared to the other treatment arm(s). These same characteristics were observed also in the analysis of the clinical trial datasets. The difference in exacerbation treatment effect estimates between the joint frailty and standard models was largest in Datasets C-E, which were the datasets showing the biggest separation between treatment arms in terms of treatment discontinuations. The absolute difference in treatment effect estimates in these datasets were between 4 and 15 percentage points, which can be regarded as clinically relevant.

In our covariate search, we identified prognostic factors of exacerbation risk that all have been previously established [31–35]. However, their impact has – to our knowledge – never been quantified using a joint frailty model, and our analysis further supports their value in predicting exacerbation risk. The use of the joint frailty model also allowed us to include the time-varying effect of seasonality in our analysis, confirming a higher risk of exacerbations during the winter months and a lower risk during summer [28].

Interestingly, several of the covariates associated to exacerbation risk were also associated to treatment discontinuation risk, supporting our finding that the two risks are associated. Including prognostic factors in the model, however, resulted in a relatively small reduction of the *frailty* variance, which is in line with the general understanding that known risk factors explain only a small part of COPD patient heterogeneity [34, 36]. It also highlights the importance of considering heterogeneity in the statistical analysis, even after adjusting for known covariates.

Our analysis is limited by the fact that we, according to data re-use rules and consent reasons, did not have access to data from all patients included in the original trials. Of particular note is that treatment discontinuation rates were higher in the original trials compared to the rates in our analysis datasets (see Table 2). As shown in our simulation study, an increased rate of treatment discontinuations can contribute to an increased bias in exacerbation treatment effect estimates of models disregarding the association between exacerbations and treatment discontinuations. Therefore, differences in treatment effect estimates between the joint frailty model and the shared frailty and negative binomial models would likely have been even more pronounced had we been able to use the original trial datasets.

While our analysis clearly demonstrates an overall association between the risks of exacerbation and treatment discontinuation, it is also limited by the fact that we have treated all discontinuations the same regardless of reason (e.g. adverse events, disease worsening, or lost to follow-up). Further work may be done to exclude discontinuations known to be unrelated to the disease or trial (although such a classification can sometimes be difficult or not possible due to missing information). Particularly, this may impact the analysis of the roflumilast data, where GI adverse events lead to discontinuations – predominantly during the first month of treatment – and where the association to exacerbation risk can be questioned. Indeed, the overall risk association was estimated to be weaker in Datasets D-E. Further work could also include exploring alternative model structures to describe the association between exacerbation and discontinuation risks.

## Conclusions

We have identified a significant association between the risks of exacerbation and early treatment discontinuation in COPD clinical trials and show that treatment discontinuations per se contribute important information on exacerbation outcomes – results we believe add significant value to the discussion on missing data in COPD and clinical trials in general [6, 10]. We regard the joint frailty model as a useful approach to study the impact of discontinuations on exacerbation treatment effect estimates and recommend it to be used as a complement to standard analyses in COPD clinical trials, e.g. when differentiating the clinical benefit of an effective and less effective treatment (assay sensitivity) [37].

## Supplementary information

**Additional file 1.** Additional details on datasets, statistical methods, simulation study, and additional results from the clinical trial data

## Data Availability

De-identified patient data underlying the findings described in this manuscript can be requested in accordance with AstraZeneca’s data sharing policy described at https://astrazenecagrouptrials.pharmacm.com/ST/Submission/Disclosure. The R code that supports the findings of this analysis is available on request from the corresponding author.

## Abbreviations

AG: Andersen-Gill, bid: twice daily
BUD: Budesonide
CAT: Chronic obstructive pulmonary disease Assessment Test™
CI: Confidence interval
COPD: Chronic obstructive pulmonary disease
DPI: Dry powder inhaler
FEV_1_: Forced expiratory volume in 1 s
FM: Formoterol
GI: Gastrointestinal
HR: Hazard ratio
ICS: Inhaled corticosteroid
LABA: Long-acting beta agonist
LAMA: Long-acting muscarinic receptor antagonist
MAR: Missing at random
PBO: Placebo
pMDI: Pressurized metered-dose inhaler
PROs: Patient reported outcomes
RFL: Roflumilast
SABA: Short-acting beta agonists
SE: Standard error
SGRQ: St. George’s Questionnaire
SOC: Standard of care
US: United States
