# Supplementary material for: Improving the evaluation of COPD exacerbation treatment effects by accounting for early treatment discontinuations: a post-hoc analysis of randomized clinical trials
Source: Respir Res. 2020 Jun 22;21:158. doi: 10.1186/s12931-020-01419-8 (PMC7310001; doi:10.1186/s12931-020-01419-8)
Supplement: Supplementary file 1 — Additional file 1. Additional details on datasets, statistical methods, simulation study, and additional results from the clinical trial data [file 12931_2020_1419_MOESM1_ESM.pdf]

Improving the evaluation of COPD exacerbation treatment effects by accounting for early treatment discontinuations: a post-hoc analysis of randomized clinical trials

Agnieszka Król, Robert Palmér, Virginie Rondeau, Stephen Rennard, Ulf G Eriksson, Alexandra Jauhiainen

Supplementary material

S1 Study populations and inclusion criteria

The trials A-E [1, 2, 3, 4, 5] were conducted worldwide, in 237 centres in the United States, Europe and Mexico (A); 180 centres in the United States, Central and South America (B); 194 centres in the United States, Czech Republic, Poland, the Netherlands and South Africa (C); 203 centres in Europe and several countries worldwide: Australia, Canada, Israel, Republic of Korea, South Africa (D); 380 centres in 17 countries in Europe, North, Central and South America and Asia. Studies A-C had similar inclusion criteria to select patients with moderate to severe COPD with a history of exacerbations. Eligible patients were 40 years or older, current or ex-smokers with smoking history of ≥10 pack-years diagnosed with severe airflow limitation confirmed by pre-bronchodilator forced expiratory volume in 1 second (FEV1) of ≤50% predicted and pre-bronchodilator FEV1/forced vital capacity (FVC) ratio <70%. Patients had to also have a history of at least one COPD exacerbation requiring treatment in the 12 months before the first study visit.

There were a few differences in the inclusion criteria for studies E and D, which aimed at recruiting patients with severe COPD. The included individuals had to have a smoking history of ≥20 pack-years, post-bronchodilator FEV1 of ≤50% predicted and post-bronchodilator FEV1/FVC ratio <70%. Eligible patients had also at least two COPD exacerbations in the one year period before the trial. Moreover, in study D, patients had to be treated with an inhaled corticosteroid (ICS) and long-acting β_2-agonist (LABA) combination for 12 months prior to the study and have a total sum of cough and sputum scores of 14 or higher during the week preceding the randomization data (0-4 scores reported daily). In study E, patients had to have been receiving ICS/LABA daily for at least 3 months prior to screening.

Patients in the trials could be discontinued from study treatment at any time. Reasons for early treatment discontinuations observed in respective datasets are presented in Table S1.

S2 Joint frailty model for recurrent episodes and a terminal event

For a patient i, $i = 1, \cdots, N$ we observe time of a terminal event (time to early treatment discontinuation), $T_i = \min(T_i^*, C_i)$ which is the minimum of time to the true event from the beginning of the study, T_i^*, and the time of the right-censoring, C_i which is assumed to be non-informative and corresponds to the case when, for a given patient, an early treatment discontinuation was not observed, i.e. the individual completed the study. We denote the observed starting times

Dataset	A N (%)	B N (%)	C N (%)	D N (%)	E N (%)
No. of early treatment discontinuations	392	221	235	180	421
**Reason for early treatment discontinuation**					
Adverse event[a]	253 (64.5%)	128 (57.9%)	143 (60.9%)	76 (42.2%)	188 (44.7%)
Protocol violation	42 (10.7%)	19 (8.6%)	17 (7.2%)	0	84 (20.0%)
Deterioration or no response	NA	NA	NA	26 (14.4%)	21 (5.0%)
Death	0	0	0	27 (15.0%)	0
Physician/Sponsor decision[b]	0	0	0	23 (12.8%)	41 (9.7%)
Subject lost to follow-up	46 (11.7%)	24 (10.9%)	25 (10.6%)	12 (6.7%)	18 (4.3%)
Other reasons	51 (13.1%)	50 (22.6%)	49 (20.9%)	16 (8.9%)	69 (16.3%)
Missing information	0	0	1 (0.4%)	0	0

NA - category as such not included in the study protocol

[a] In datasets A-C, early treatment discontinuation from the study due to deterioration of COPD was recorded as "adverse event"

[b] "Sponsor decision" applies only to dataset E and means that the sites were shut down

Table S1: Reasons for early treatment discontinuation in the analyzed datasets. Percentages in the different discontinuation categories are based on the total number of discontinuations in each dataset. Among the individuals who discontinued treatment early in the trials, there were also patients who stopped treatment as they were not willing to continue and subsequently withdrew their consent. These patients are not presented here as their data was not available due to data re-use rules.

of the recurrent episodes $T^s_{ij} = \min(T^{s*}_{ij}, C_i, T^*_i)$, $j = 1, \cdots, n_i$, with T^{s*}_{ij} the true time of the start of the episode (exacerbation). Analogically, we denote the observed ending time of the recurrent episodes $T^e_{ij} = \min(T^{e*}_{ij}, C_i, T^*_i)$. We define the indicators of the events, $\delta_i = I_{\{T_i = T^*_i\}}$, for early treatment discontinuations, and $\delta_{ij} = I_{\{T^s_{ij} = T^{s*}_{ij}\}}$, for exacerbations. We assume, for all individuals, $T^e_{ij} \leq T_i$, $j = 1, \cdots, n_i$.

Let $N^s_i(t) = \sum_{j=1}^{\infty} I_{\{T^{s*}_{ij} \leq t\}}$ and $N^e_i(t) = \sum_{j=1}^{\infty} I_{\{T^{e*}_{ij} \leq t\}}$ define counting processes that represent the number of observed event starts and endings before individual i was completed or withdrew from the study since the study entry. Denote by $Y^{ex}_i(t) = I_{\{N^s_i(t-) = N^e_i(t-)\}}$ the at-risk process whether the previous exacerbation has terminated. The joint frailty model is expressed as the conditional hazard functions for recurrent and terminal events considering repeated episodes and is defined by [6]:

$$\begin{cases} r_{ij}(t|u_i) = Y^{ex}_i(t) u_i r_0(t)\ \mathrm{e}^{\boldsymbol{X}_{ex,ij}{}^\top \boldsymbol{\beta}_{ex}} \\ \lambda_i(t|u_i) = u_i^{\alpha} \lambda_0(t)\ \mathrm{e}^{\boldsymbol{X}_{ed,i}{}^\top \boldsymbol{\beta}_{ed}} \end{cases} \tag{1}$$

where $\boldsymbol{X}_{ex,ij}$ are covariates for the recurrent event process and $\boldsymbol{X}_{ed,i}$ are covariates for the

terminal event process. Parameters $\boldsymbol{\beta}_{ex}$ and $\boldsymbol{\beta}_{ed}$ are the related regression coefficients. Shared frailty terms u_i are assumed to be independent of each other and identically distributed under a gamma distribution, $u_i \sim \Gamma(1/\theta, 1/\theta)$. The variance θ reflects the population heterogeneity, the intensity of the association between the recurrent events and the terminal event as well as the correlation between the recurrent events of the same subject. Finally, the parameter of association α determines the intensity and the sign of influence of the recurrent events on the terminal event.

In a joint model for recurrent events and a terminal event, for subject i at each rank j we observe $\{T^s_{ij}, T^e_{ij}, \delta_{ij}, T_i, \delta_i\}$. Denote by $\boldsymbol{\xi} = (r_0(\cdot), \lambda_0(\cdot), \boldsymbol{\beta}^\top_{ex}, \boldsymbol{\beta}^\top_{ed}, \theta, \alpha)^\top$ the vector of parameters to estimate. We assume that the processes of recurrent and terminal events are independent of each other given the frailty u_i. Thus, the joint individual marginal likelihood can be expressed by:

$$\begin{aligned} L_i(\boldsymbol{\xi}) &= \int_{u_i} \prod_{j=1}^{n_i} \left(f_{T^{ex}|u_i}(T^s_{ij}, T^e_{ij}, \delta_{ij}|u_i; \boldsymbol{\xi}) \right) f_{T^{ed}|u_i}(T_i, \delta_i|u_i, \boldsymbol{\xi}) f_{u_i}(u_i; \boldsymbol{\xi}) du_i \\ &= \int_{u_i} \prod_{j=1}^{n_i} \left((r_{ij}(T^s_{ij}|u_i))^{\delta_{ij}} \exp\left[-\int_{T^e_{i(j-1)}}^{T^s_{ij}} r_{ij}(t|u_i) dt \right] \right) \\ &\times (\lambda_i(T_i|u_i))^{\delta}_i \exp\left[-\int_0^{T_i} \lambda_i(t|u_i) dt \right] f_{u_i}(u_i; \boldsymbol{\xi}) du_i, \end{aligned}$$

where functions $f_{T^{ex}|u_i}(\cdot|\cdot)$ and $f_{T^{ed}|u_i}(\cdot|\cdot)$ denote density functions of recurrent events and the terminal event, respectively, and $f_{u_i}(\cdot)$ is the density function of the frailty term.

The inference of the model uses the approach of penalized maximum likelihood estimation with cubic M-splines, polynomial functions of order 3 [7], for the approximation of the baseline risk functions. The penalization of the log-likelihood is performed for the smooth estimation of baseline hazard functions. The penalty terms include second derivatives of the baseline risk functions and smoothing parameters that control the trade-off between the smoothness and fit to the data. The smoothing parameters can be chosen using appropriate automatic procedures on reduced models [8].

The integral in the marginal likelihood has no analytical solution and we approximate it using the Gauss-Legendre quadrature. In order to provide accurate estimates we place the quadrature points in regions that can be problematic when the gamma distribution for the frailty has a high variance ($\theta > 1$). We use 50 points in the region (0,0.001), 50 points in (0.001, 1), 20 points in (1,10) and 20 points in (10,50). The penalized log-likelihood is maximized using the Marquardt algorithm. The standard errors of the estimated parameters are obtained from the inverse Hessian matrix of the penalized log-likelihood.

The baseline hazards are estimated in the model using the approximation of cubic M-splines. In this approach, smooth hazard functions with low local variations are assumed and this is obtained by introducing penalized maximum likelihood estimation (MPLE). In the MPLE, the log-likelihood of the model is penalized by term that have large values for rough functions. The smoothing parameters that control the trade-off between the fit to the data and smoothness, can be chosen using appropriate automatic procedures on the reduced models [8].

S3 Negative binomial model

The negative binomial model can be parametrized in a number of different ways. With the mean parametrization as is used in the R implementation of the glm.nb function, the density function is defined as

$$f(k;\mu,\theta) = \frac{\Gamma(k+\theta)}{k!\Gamma(\theta)}\left(\frac{\theta}{\mu+\theta}\right)^{\theta}\left(\frac{\mu}{\mu+\theta}\right)^{k}.$$

Here, k is the outcome (in our case the number of events), μ is the mean (number of events), and θ is a parameter related to the (excess) variance modeled compared to a simple Poisson distribution. If X has a negative binomial distribution under this parametrization we have that

$$V(X) = \mu + \frac{1}{\theta}\mu^2 = \mu + \delta\mu^2$$

where we refer to δ as the dispersion parameter. This parameter is tabulated in table S3 for the negative binomial models studied.

S4 Software

The joint frailty and shared frailty models were all estimated using the R package frailtypack [11]. The negative binomial models were estimated with the function glm.nb from the R package MASS [12]. The LASSO regression was performed using the R package glmnet [13].

S5 Simulation study

The main purpose of the simulation study was to investigate the impact of differential early treatment discontinuation, either positively or negatively related to therapy and/or medications, on the estimation of the treatment effect on the risk of recurrent exacerbations.

S5.1 Data generation

We generated recurrent event times and terminal event times from a joint frailty model assuming the processes being positively correlated. The generated datasets were similar to the clinical ones; we assumed a study length of one year (fixed right-censoring) with two treatment groups and 800 individuals in total. Firstly, for an individual, we generated the frailty parameter from a gamma distribution, then we randomly assigned the individual to the treatment group and we used these values to generate the time to early treatment discontinuation using exponential baseline hazard functions. If the generated time to early treatment discontinuation was larger than one year, the individual was considered as "censored", i.e. he/she completed the study and the early treatment discontinuation was not observed. Then, considering the generated time to terminal event, we generated recurrent event times up to the time of the end of observation. We assumed the hazard ratio of treatment for recurrent exacerbations equal to 0.6.

S5.2 Scenarios and models

We investigated the bias of treatment effect on exacerbations assuming different effects of treatment on early discontinuations. In particular, we considered hazard ratios for discontinuation

related treatment effects equal to 0.5, meaning that individuals tended to discontinue more in the control group, HR = 1, which means no differential early treatment discontinuation, and HR = 2 that corresponded to the case of more early withdrawals in the treatment group. For each scenario we also considered different rates of exacerbations and early treatment discontinuations. We controlled the rates of events by adjusting the exponential distribution parameter for the baseline hazards. Finally, we investigated the bias by considering different combinations of strong ($\alpha = 1.5$) and weak association ($\alpha = 0.8$) between the recurrent and terminal event processes and high ($\theta = 2$) and moderate ($\theta = 0.8$) variance of the frailty term.

The simulations results for each scenario were obtained through 1000 replicates of 800 individuals. We estimated the shared frailty and joint frailty models with an assumed gamma distribution for the frailty term and applied splines with 4 knots distributed using equidistant knots. The penalty terms were found for each dataset using the automatic cross-validation procedure in the shared frailty model for exacerbations. In the joint model, we used the same penalty for recurrent episodes and for terminal events, we used the penalty obtained from a Cox model for early treatment discontinuations. In case of non-convergence, we changed the value of the penalty term (by multiplying by 10 values smaller than 100 and dividing by 10 values larger than 100) until convergence was reached.

S5.3 Results

In all scenarios, we observed biased estimates of the treatment effect on the risk of exacerbations using shared frailty models that ignore the process of early treatment discontinuations (Figure S1, the right bottom panel corresponds to the results presented in Figure 3 in the main text). When applying the joint frailty model to the same generated datasets, we found unbiased results (Figure S2).

With the shared frailty model, we observed underestimation of the treatment effect on the risk of exacerbations when more patients withdraw from the study in the control group (HR = 0.5 for treatment effect on the risk of early discontinuations). On the other hand, the treatment effect on the risk of exacerbations was overestimated for HR on early treatment discontinuations equal to 2 (similar to trends observed in datasets D and E), when the treatment is associated with increased risk of early discontinuations. Finally, when there were no differential early treatment discontinuations, i.e. HR of the treatment effect on the risk of discontinuations equaled 1, we observed small bias in the estimates of the effect of treatment on the risks of exacerbations in the shared frailty model.

We observed a larger bias in scenarios with the strong association and high variance compared to the scenarios of the weak association and moderate variance. When considering the rates of events on the magnitude of the bias of the treatment effect, we found that the number of exacerbations observed per patient had less influence than the rate of observed early treatment discontinuations in the data. In the case, when there were many discontinuations (40%), we found larger average bias than in the scenario with smaller number of discontinuations (25%). Indeed, when we have many early treatment discontinuations in the data, the average time of follow up is shorter and we are less able to correctly estimate the parameters. On the other hand, the results showed that even if the rate of discontinuations is not larger than 25%, as it is the case in our application, we are still able to observe the bias of the effect of treatment on the risk of exacerbations if the process of early treatment discontinuations is ignored.

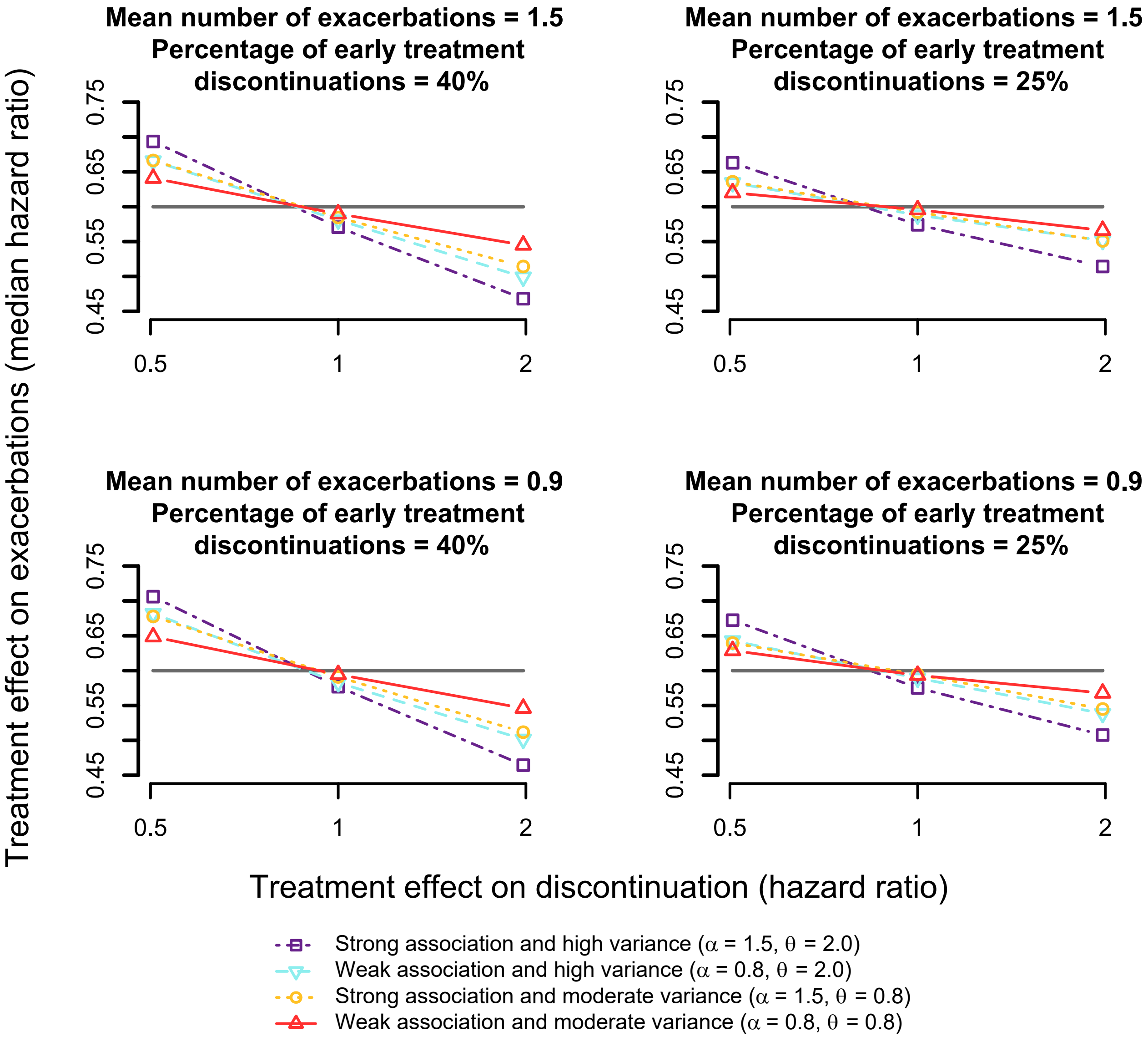

Figure S1: Bias of treatment effect on the risk of exacerbations using reduced shared frailty model that ignores the process of early treatment discontinuations in the simulation study in scenarios of different strength of association between the generated processes. Different points on the plot represent scenarios with different rates of exacerbations and early treatment discontinuations in the data.

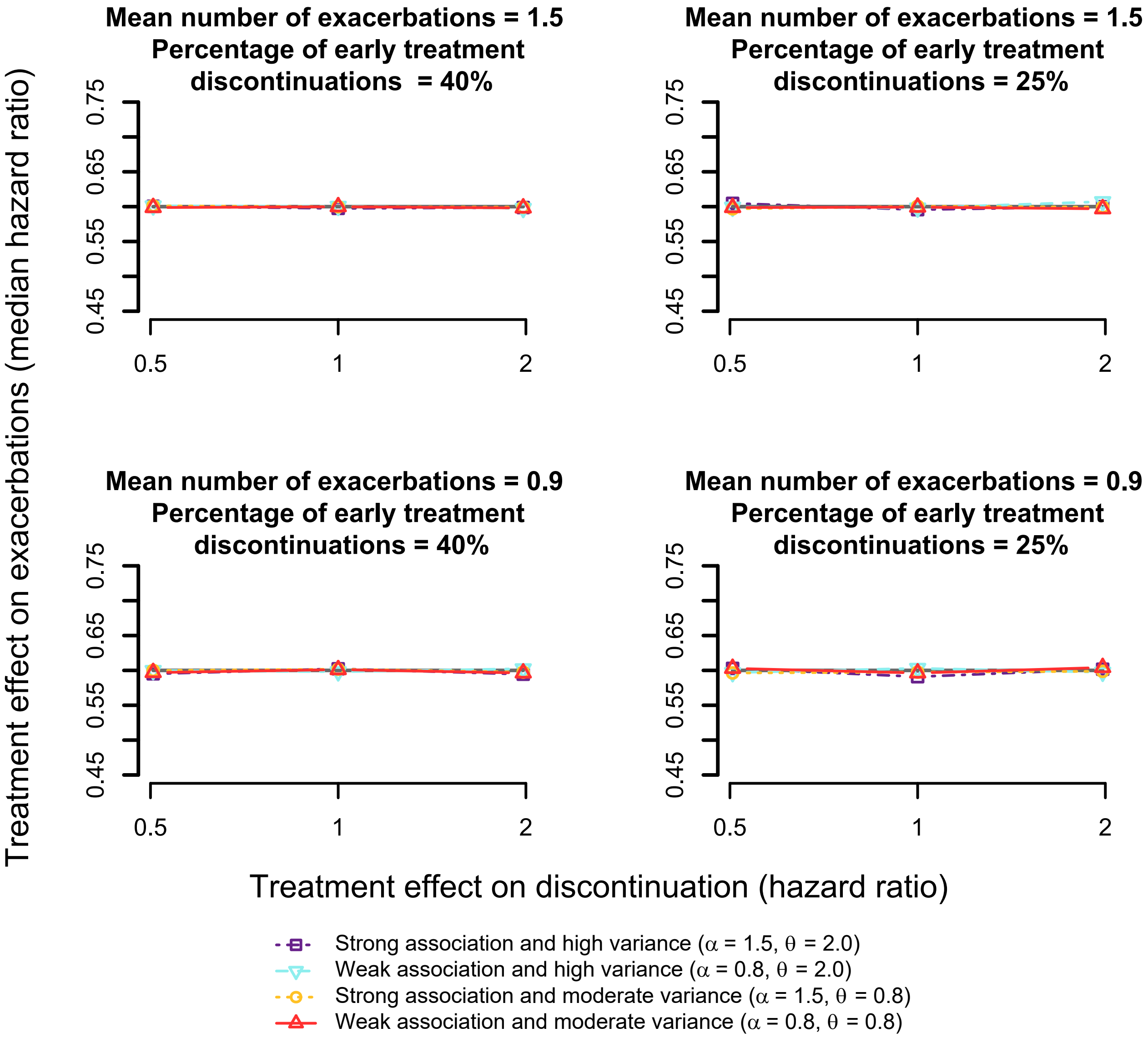

Figure S2: Bias of treatment effect on the risk of exacerbations using joint frailty model that includes the process of early treatment discontinuations in the simulation study in scenarios of different strength of association between the generated processes. Different points on the plot represent scenarios with different rates of exacerbations and early treatment discontinuations in the data.

	Negative binomial model<br>Dispersion parameter	Shared frailty model<br>Frailty variance
	Estimate (SE)	
Dataset A	1.87 (0.04)	1.85 (0.16)
Dataset B	0.81 (0.16)	0.79 (0.10)
Dataset C	2.12 (0.05)	2.15 (0.25)
Dataset D	1.35 (0.06)	1.36 (0.11)
Dataset E	0.75 (0.11)	0.75 (0.06)

SE - standard error

Table S2: Estimates of the dispersion and frailty variance parameters in reduced models for moderate/severe exacerbations in all datasets.

S6 Analysis of clinical trial data

S6.1 Models applied to COPD data with treatment as the only covariate

For the COPD datasets we estimated models with only treatment effect as a covariate. For the shared frailty and joint frailty models, we used percentile splines (the intervals for splines were divided in a way to obtain the same number of events in each interval) for estimating the baseline hazard functions with the number of knots chosen to ensure the best goodness-of-fit to the data (dataset A: 13, B: 12, C: 12, D: 15, E: 14). Random effects were assumed to be distributed according to a gamma distribution so that the models would be similar to the rate analysis using negative binomial models. However, a sensitivity analysis of the frailty distribution showed that the estimations and goodness-of-fit (using an approximated likelihood cross-validation criterion, LCV [9]) were almost identical for both gamma and log-normal joint frailty models.

The exacerbation hazard ratios in all the models as well as association parameters in the joint frailty model are presented in the main manuscript (Tables 3 and 4). Estimates of the frailty variance in the shared frailty model and dispersion parameters in the negative binomial model are presented in Table S2. Early treatment discontinuation hazard ratios in the joint frailty model are presented in Table S3.

For comparison with pooled data analysis with covariates, results of the joint frailty model for pooled dataset with no covariates (only treatment and study effect) are presented in Table S4.

S6.2 Joint models with covariates applied to pooled COPD datasets

The analysis that included demographic and disease related prognostic factors as covariates in addition to treatment was performed on pooled datasets. The variables are listed in Table S5. We combined datasets with similar treatments and similar design; from datasets A, B and C we created pooled A-C and from datasets D and E we formed pooled D-E.

The covariates were selected for the analysis using a combination of an automatic procedure and clinical knowledge. Firstly, the LASSO (least absolute shrinkage and selection operator)

	Treatment effect on early treatment discontinuations	
	Hazard ratio	95% CI
Dataset A		
BUD/FM 320 vs PBO	0.73	(0.51 - 1.04)
BUD/FM 160 vs PBO	0.84	(0.59 - 1.19)
FM vs PBO	1.00	(0.71 - 1.41)
Dataset B		
BUD/FM 320 vs FM	0.85	(0.58 - 1.26)
BUD/FM 160 vs FM	0.87	(0.59 - 1.28)
Dataset C		
BUD/FM 320 vs PBO	0.51	(0.28 - 0.91)
BUD/FM 160 vs PBO	0.43	(0.24 - 0.79)
BUD 320 + FM vs PBO	0.52	(0.29 - 0.94)
BUD 320 vs PBO	0.75	(0.43 - 1.31)
FM vs PBO	0.93	(0.54 - 1.61)
Dataset D		
SOC + RFL vs SOC	1.79	(1.29 - 2.49)
Dataset E		
SOC + RFL vs SOC	1.54	(1.25 - 1.89)

CI - confidence interval

Table S3: Results of the joint model estimation for early treatment discontinuations for Datasets A-E. Only treatment included as covariate.

	Exacerbations (moderate/severe)		Early treatment discontinuations	
	Pooled A-C HR (95% CI)	Pooled D-E HR (95% CI)	Pooled A-C HR (95% CI)	Pooled D-E HR (95% CI)
**Treatment**				
BUD/FM 320 vs FM	0.67 (0.57 - 0.78)		0.72 (0.56 - 0.91)	
BUD/FM 160 vs FM	0.68 (0.58 - 0.80)		0.77 (0.60 - 0.98)	
BUD 320 + FM vs FM	0.55 (0.40 - 0.75)		0.64 (0.41 - 1.02)	
BUD 320 vs FM	0.70 (0.51 - 0.95)		0.94 (0.61 - 1.46)	
PBO vs FM	1.03 (0.85 - 1.25)		0.99 (0.75 - 1.30)	
SOC + RFL vs SOC		0.89 (0.81 - 0.99)		1.59 (1.32 - 1.91)
**Trial effect**				
A vs C	0.92 (0.79 - 1.08)		1.05 (0.83 - 1.32)	
B vs C	1.21 (1.02 - 1.44)		0.96 (0.73 - 1.24)	
E vs D		1.05 (0.94 - 1.17)		1.48 (1.21 - 1.81)

**Association parameters**				
	Pooled A-C		Pooled D-E	
	Estimate	SE	Estimate	SE
Frailty variance (θ)	1.56	0.08	0.94	0.05
Association (α)	1.22	0.10	0.86	0.12

HR - hazard ratio, CI - confidence interval, SE - standard error

Table S4: Results of the joint model estimation for pooled datasets. Only treatment and study included as covariates.

Category	Variables	Remarks
Demographics	age in years, sex, weight, race, country, smoking status (former/current), smoking history (no. of pack-years)	
Disease severity	years since COPD diagnostics, exacerbation history (no. exacerbations/observation time in years), past or present coexistence of cardiovascular disease, use of ICS $\geq$1 month before the study, use of bronchodilators $\geq$1 month before the study, use of LAMA	values at baseline
Laboratory variables	eosinophils, neutrophils, basophils, monocytes, lymphocytes concentration, bilirubin concentration	values at baseline
Spirometry measures	pre- (A-C)/post-bronchodilator (D-E) FEV1, reversibility	average values from the run-in period
Patient reported outcomes	breathlessness, sleep, cough, sputum troubles, wheeze (0-4 scores), no. of rescue medication inhalations during a day (total of day and night)	average values from the run-in period
SGRQ score (A-C trials)	total score of symptoms, activity and impact (0-100 score)	average values from the run-in period
CAT score (D-E trials)	total score, i.e. sum of cough, mucus, tightness of chest, breathlessness, activities limitation, confidence to leave home, sleep, energy (0-40 score)	value at baseline

Table S5: Baseline variables considered in the analysis.

Cox regression [10] was performed for simplified models: time to first exacerbation and time to early treatment discontinuation applied to separate datasets. Four methods for finding optimum penalty were applied: 10-fold cross-validation (CV) rule, one standard error 10-fold CV rule, BIC rule and bootstrap (B=1000) BIC rule. Then, we created a set of covariates (including treatment) for datasets A-C if the covariates were selected by at least two different methods in at least two datasets. For datasets D-E, the covariates were chosen if they were selected in at least two methods. The covariate sets were complemented by variables that, although not selected by the LASSO procedure, were found to be clinically meaningful. The final sets of covariates for each study was found using stepwise backward selection with joint frailty models on each pooled dataset. In this step we also added the time-varying seasonality covariate. To facilitate the model selection, all continuous variables were standardized. The respective steps of the covariate selection are presented in Figure S3 and the specifications of the final set of variables can be found in table 5 of the main text.

In the final model for pooled A-C we did not use the laboratory variable (neutrophils, significant effect on exacerbation risk, p-value = 0.04) even though it was chosen in the backward selection procedure as there were missing values for an important number of patients (48 in dataset A, 38 in B and 36 in C) and imputing values would introduce additional uncertainty to the estimates. Summary of all the included covariates is presented in Table S6.

We applied splines for the approximation of baseline hazard functions using the approach of percentiles. Using graphical assessment of the estimated baseline hazard functions and fit to the data using LCV, we found that 13 knots were enough to well approximate the functions in both pooled A-C and D-E datasets. The plots of the estimated baseline hazard risks are shown in Figure S4. We observed hazard functions for exacerbations with quite regular periods of increased and decreased risks during the follow-up. The pattern of periods of increased risks was similar for exacerbations and early treatment discontinuations but the increase of the risk of discontinuation was smaller compared to exacerbations.

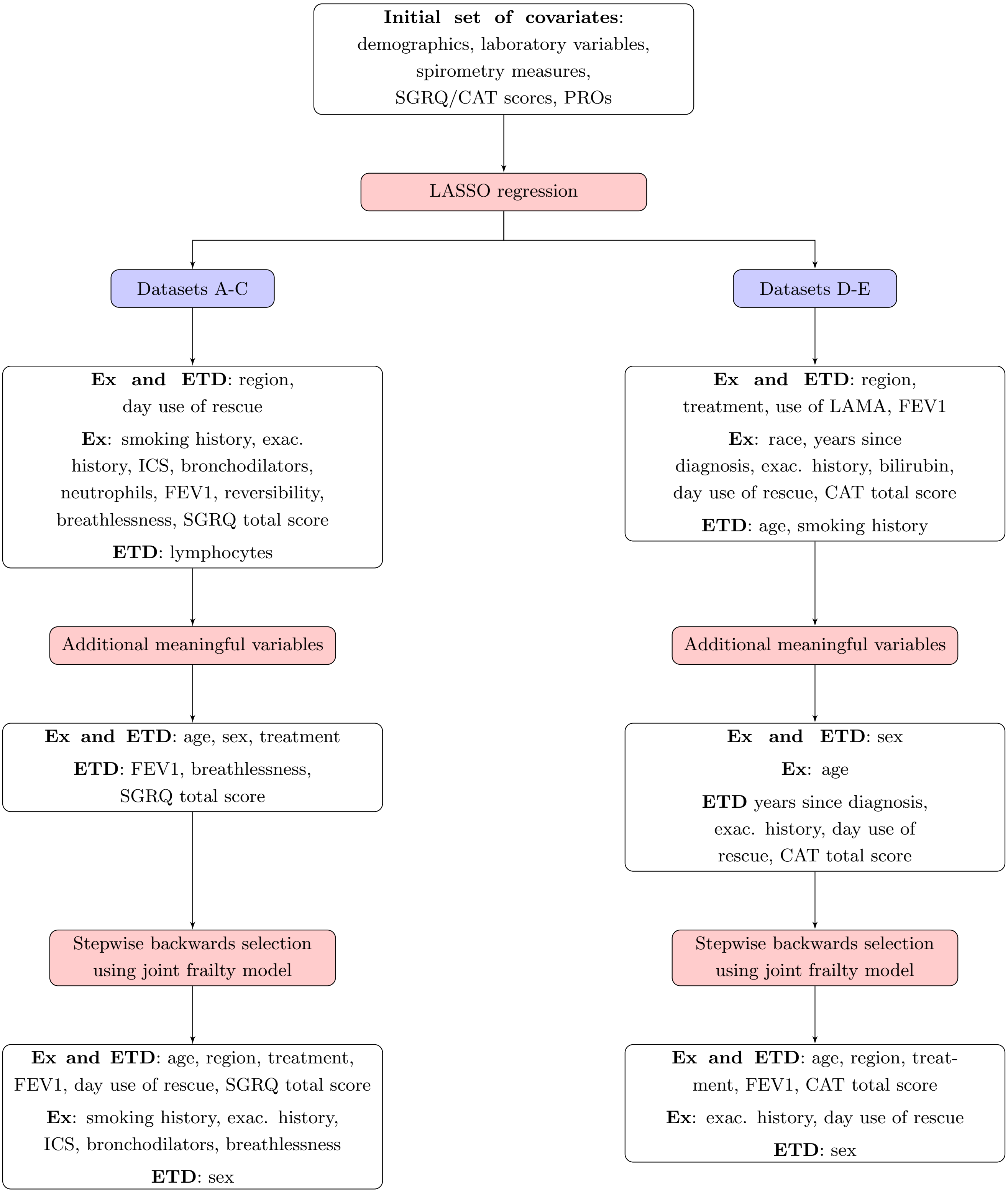

Figure S3: Flow chart of covariate selection process for the joint models analysis (**Ex** - exacerbation risk process, **ETD** - early treatment discontinuation risk process).

	Pooled A-C		Pooled D-E	
	N/Median	%/Range	N/Median	%/Range
No. of patients*	4355		3058	
**Demographic characteristics**				
Sex (Male)	2847	65%	2154	70%
Age, years	63	40 – 90	64	40 – 89
Region				
US	1890	43%	807	26%
Western Europe	409	9%	414	14%
Eastern Europe	1286	30%	887	29%
Rest of the World	770	18%	950	31%
**Treatment**				
BUD/FM 320	1070	25%	–	–
BUD/FM 160	1060	24%	–	–
BUD 320 + FM	271	6%	–	–
BUD 320	252	6%	–	–
FM	1042	24%	–	–
PBO	660	15%	–	–
SOC + RFL	–	–	1502	49%
SOC	–	–	1556	51%
**Disease severity**				
History (no exac./observation years)	1	0 – 12	2	1 – 9
Use of ICS (Yes)	2296	53%	–	–
Use of bronchodilators (Yes)	2317	53%	–	–
FEV1**	0.99	0.24 – 2.99	0.97	0.22-2.11
SGRQ total score (0-100)	54.98	3.74 – 99.22	–	–
Breathlessness score (0-4)	2.07	0 – 4	–	–
CAT total score (0-40)	–	–	19.00	0 – 40
Rescue medication (no of puffs/day)	3.50	0 – 42.20	3.45	0 – 39.50

BUD - budesonide, FM - formoterol, RFL - roflumilast, SOC - standard of care

PBO - placebo

* Numbers after removing individuals with missing values of the covariates.

** Pre-bronchodilator FEV1 in A-C and post-bronchodilator FEV1 in D-E.

Table S6: Patient characteristics in the analyzed studies for variables included in the full joint frailty models.

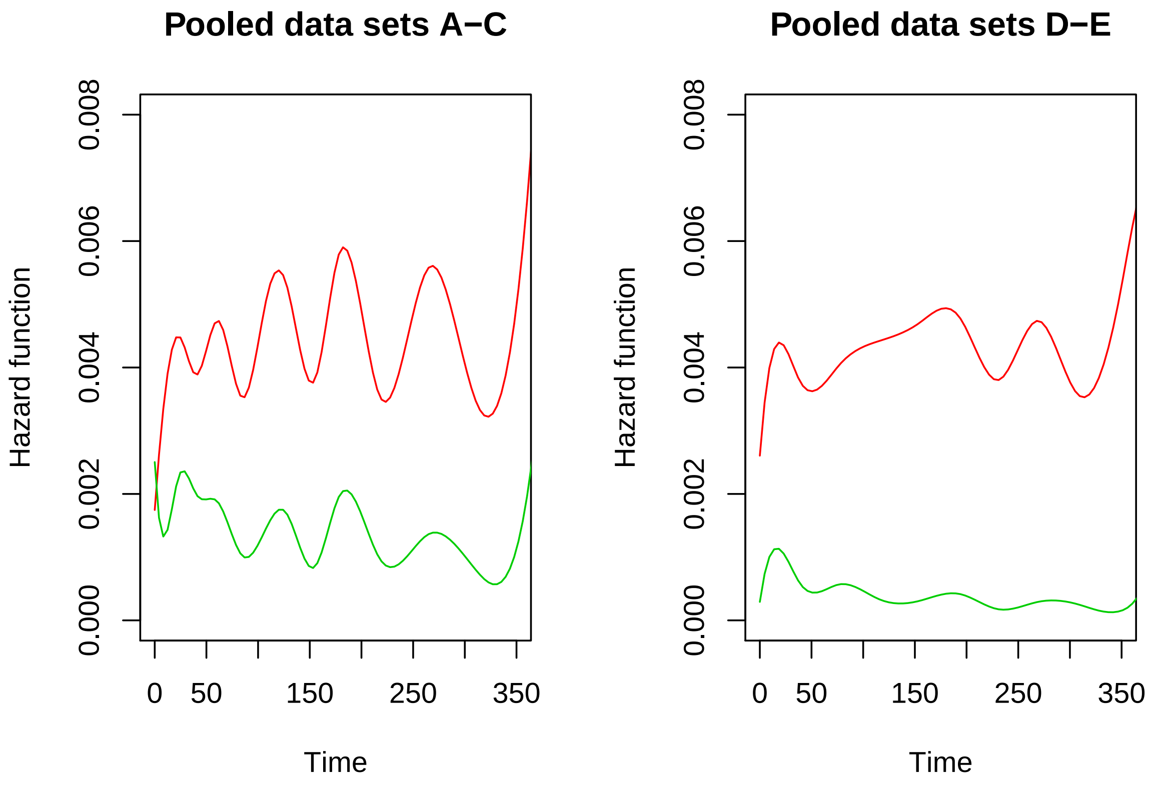

Figure S4: Estimated baseline hazard functions of the full joint frailty models for pooled datasets (output from the package *frailtypack*). Red lines correspond to baseline hazard risks of recurrent moderate/severe exacerbations and green lines to early treatment discontinuations.

References

[1] Rennard, S. I., Tashkin, D. P., McElhattan, J., Goldman, M., Ramachandran, S., Martin, U. J., & Silkoff, P. E. (2009). Efficacy and tolerability of budesonide/formoterol in one hydrofluoroalkane pressurized metered-dose inhaler in patients with chronic obstructive pulmonary disease. *Drugs*, 69(5), 549-565.

[2] Sharafkhaneh, A., Southard, J. G., Goldman, M., Uryniak, T., & Martin, U. J. (2012). Effect of budesonide/formoterol pMDI on COPD exacerbations: a double-blind, randomized study. *Respiratory medicine*, 106(2), 257-268.

[3] Tashkin, D. P., Rennard, S. I., Martin, P., Ramachandran, S., Martin, U. J., Silkoff, P. E., & Goldman, M. (2008). Efficacy and safety of budesonide and formoterol in one pressurized metered-dose inhaler in patients with moderate to very severe chronic obstructive pulmonary disease. *Drugs*, 68(14), 1975-2000.

[4] Martinez, F. J., Calverley, P. M., Goehring, U. M., Brose, M., Fabbri, L. M., & Rabe, K. F. (2015). Effect of roflumilast on exacerbations in patients with severe chronic obstructive pulmonary disease uncontrolled by combination therapy (REACT): a multicentre randomised controlled trial. *The Lancet*, 385(9971), 857-866.

[5] Martinez, F.J., Rabe, K.F., Sethi, S., Pizzichini, E., McIvor, A., Anzueto, A., Alagappan, V.K., Siddiqui, S., Rekeda, L., Miller, C.J., Zetterstrand, S., Reisner, C., & Rennard, S.I. (2016). Effect of roflumilast and inhaled corticosteroid/long-acting β_2-agonist on chronic obstructive pulmonary disease exacerbations (RE^{2}SPOND). A randomized clinical trial. *Am. J. Respir. Crit. Care Med.*, 194(5), 559-67.

[6] Rondeau, V., Mathoulin-Pelissier, S., Jacqmin-Gadda, H., Brouste, V., & Soubeyran, P. (2007). Joint frailty models for recurring events and death using maximum penalized likelihood estimation: application on cancer events. *Biostatistics*, 8(4), 708-721.

[7] Ramsay, J. O. (1988). Monotone regression splines in action. *Statistical science*, 3(4), 425-441.

[8] Rondeau, V., Commenges, D., & Joly, P. (2003). Maximum penalized likelihood estimation in a gamma-frailty model. *Lifetime data analysis*, 9(2), 139-153.

[9] Commenges, D., Joly, P., Gegout-Petit, A, & Liquet, B. (2007). Choice between semi-parametric estimators of Markov and non-Markov multi-state models from coarsened observations. *Scandinavian Journal of Statistics*, 34(1), 33-52.

[10] Tibshirani R. (1997). The lasso method for variable selection in the Cox model. *Stat Med*, 16(4), 385-395.

[11] Król, A., Mauguen, A., Mazroui, Y., Laurent, A., Michiels, S., Rondeau, V. (2017). Tutorial in Joint Modeling and Prediction: A Statistical Software for Correlated Longitudinal Outcomes, Recurrent Events and a Terminal Event. *J Stat Softw*, 81(3), 1-52.

[12] Venables, W.N., Ripley, B.D.: Modern Applied Statistics with S, 4th edn. Springer, New York (2002). ISBN 0-387-95457-0. http://www.stats.ox.ac.uk/pub/MASS4

[13] Friedman, J., Hastie, T., Tibshirani, R.: Regularization Paths for Generalized Linear Models via Coordinate Descent. *J Stat Softw*, 33(1), 1-22.
